# Possible indirect transmission of COVID-19 at a squash court, Slovenia, March 2020: case report

**DOI:** 10.1017/S0950268820001326

**Published:** 2020-06-19

**Authors:** A. Brlek, Š. Vidovič, S. Vuzem, K. Turk, Z. Simonović

**Affiliations:** National Institute of Public Health, Regional Unit Maribor, Maribor, Slovenia

**Keywords:** COVID-19, epidemiology, outbreaks, pandemic, transmission

## Abstract

Since the beginning of the COVID-19 epidemic, there is an ongoing debate and research regarding the possible ways of virus transmission. We conducted an epidemiological investigation which revealed a cluster of five COVID-19 cases, linked to playing squash at a sports venue in Maribor, Slovenia. Acquired data raises possibility that the transmission occurred indirectly through contaminated objects in changing room or squash hall or via aerosolisation in squash hall.

## Introduction

Transmission of COVID-19 occurs mainly during close contact between people and through respiratory droplets produced during symptomatic phase, when an infected person coughs or sneezes [1]. However, there is ongoing research to identify other possible ways of transmission, such as transmission through faecal–oral route [2, 3], indirect transmission through contaminated common objects or with virus aerosolisation [4, 5]. There is also growing evidence of virus spread by asymptomatic carriers [6–9]. The aim of this report is to present a cluster of cases where transmission seems to have occurred indirectly through contaminated objects or aerosol.

## Epidemiology

Epidemiological investigation revealed a cluster of five COVID-19 cases linked to playing squash at a sports venue in Maribor, Slovenia. Index patient (person A) was travelling in Italy from 29 February to 2 March, where he most likely acquired the infection. He developed symptoms of the disease (tiredness and fatigue) on 4 March during a game of squash. Later epidemiological investigations linked four other cases of COVID-19 to the same squash hall.

Person A was playing squash with person B. They arrived at the sports venue a few minutes before 17:30. They changed their clothes in a dressing room, but they were not able to remember which dressing room they used. They began their match at 17:30 in a squash hall number one and played until 18:30, when they returned to the dressing room. Person B changed clothes and left soon after that. Person A showered and left around 18:45, but no later than 19:00. They were alone in dressing room. Person A did not report meeting anyone on his way out. Person A developed fever (>38 °C) at home later that day. He sought medical help on 5 March, when a nasopharyngeal swab was taken and reverse transcription polymerase chain reaction (RT-PCR) confirmed COVID-19 infection. We identified person B as a close contact and provided him with instructions about self-monitoring. He developed symptoms (headache, rhinorrhoea and fever) on 10 March RT-PCR confirmed the infection on 14 March.

Person C and person D also played squash at the same sports venue on 4 March. They arrived at 19:10, changed their clothes in dressing room number three, and began their match at 19:15 in hall number 1. They played until 20:00, then rested and talked with persons E and F in the hallway. After that they returned to dressing room, where person C took a shower. They both left at 20:30. Person C developed symptoms on 7 March and tested positive on 11 March. Person D developed symptoms on 8 March and tested positive on 12 March.

**Fig. 1.**
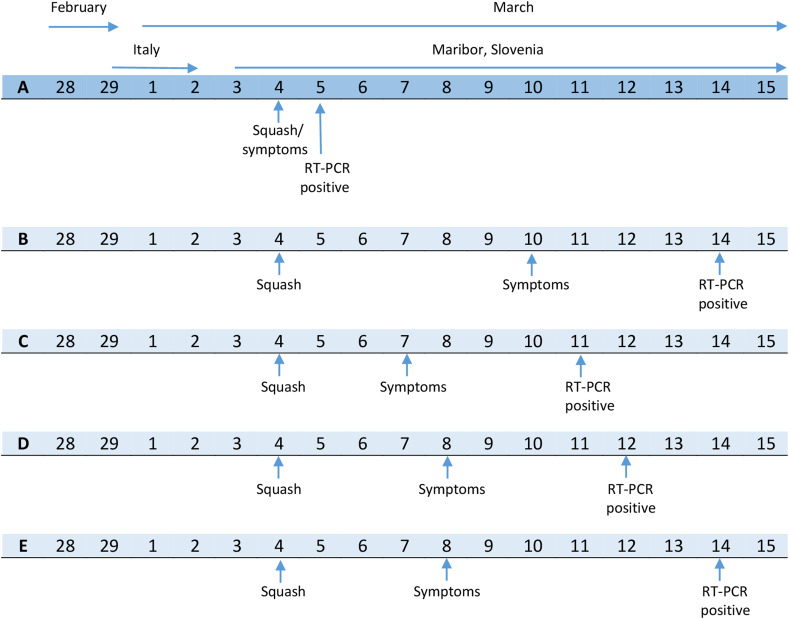
Chronology of onset and diagnosis date of five COVID-19 cases linked to playing squash at a sports venue, Maribor, Slovenia, March 2020.

Persons E and F arrived at 19:50, changed their clothes in dressing room number three, talked with persons C and D in the hallway in front of hall number 1 and began their match at 20:00. They played until 20:45, returned to the dressing room, where person E took a shower. They left at 21:00. Person E developed symptoms on 8 March and tested positive on 14 March. Person F also developed mild symptoms. Clinician instructed him to self-isolate, but did not indicate diagnostics and laboratory confirmation (Fig. 1).

The time difference between the first pair (A and B) exiting the venue and the second pair arriving (C and D) was at least 10 min, more likely around 20 min. In addition, the second pair began their match in hall 1 approximately 45 min after the first pair ended. Person E arrived an hour after person A left the sports venue, and began to play in hall 1 approximately 90 min after the first pair finished.

Unfortunately, we could not confirm if all three pairs changed in the same dressing room, but all of them reported playing in the same squash hall. None of them shared any sport equipment and had no contact with the receptionist or any other employee at the sports venue. There are five employees at the venue and none of them were symptomatic at the time, neither did they develop any symptoms later. One of them got tested, but RT-PCR came back negative. The same venue also offers different types of sport activities (basketball, soccer, fitness and badminton), but we did not identify cases linked to any other sport activity at this venue.

## Discussion

Our epidemiological investigation raises possibility that secondary cases in the cluster got the infection through indirect transmission at the sports venue.

The only plausible source of infection we were able to identify was player A. All secondary cases developed first symptoms within the incubation period (3–6 days for presented cases). None of the secondary cases had any other epidemiological link to other confirmed cases or symptomatic persons outside of squash hall. Outside the squash hall, none of them reported any other contact with one another after that day.

We cannot exclude the possibility of transmission through an unconfirmed or asymptomatic employee that works at the sports venue, because RT-PCR was not performed on all employees. However, we reduced this possibility with additional questioning of the patients and epidemiological investigation at the venue.

In the time of identifying and managing this cluster, the epidemic in Slovenia had just started and there was no community spread. Slovenia began testing for COVID-19 infection as early as 27 January and the first case was confirmed on 4 March. On 8 March (2 days before last players, cases D and E, developed symptoms), there were only 19 confirmed cases in the country and only one other confirmed case (imported) in Maribor region. Until 12 March, we did not detect community spread in Slovenia, since all cases were either imported or epidemiologically linked to other confirmed cases. We identified the first case in Maribor region without a known source of infection as late as 20 March. This shows that it is unlikely that the persons identified in the cluster were infected from different sources. However, we cannot exclude this possibility completely, because undetected community spread could have existed before those dates.

After thorough questioning of everyone involved, we concluded that persons C and D had no direct contact with the index patient (person A), who had been mildly symptomatic at the time of playing squash at the sports venue. The last pair of players (persons E and F) also denied any contact with the index patient. Because of the time difference between the game of first and last pair of players, it is highly unlikely they had any direct contact. Persons C, D and E reported direct contact with each other, but they all developed symptoms on 7 or 8 March. Because of very similar date of onset, a common source (person A) seems more plausible than transmission from person C to E and D or vice versa.

We concluded that the mode of transmission between the index patient and the secondary cases in this cluster was either through contaminated common objects or virus aerosol, since all three pairs shared the same squash hall, which is a small and confined space with poor ventilation, where strenuous physical activity is performed, during which shedding and aerosolisation of the virus could be increased [4, 10–12]. Moist and warm atmosphere coupled with turbulent air flow generated by intense physical activity could extend the lifetime of virus-bearing droplets and eventually produce residues that may stay suspended in the air for hours [13, 14]. The time difference between first and third pair of players intrigued us; however, some early studies performed in experimental settings show that virus can remain viable and infectious in aerosols for several hours and up to days on surfaces [5]. This could explain our epidemiological findings.

Clusters with epidemiological evidence of indirect transmission in shopping malls [4] and dance classes [11] have been published, but we did not find reports where transmission occurred indirectly with reported time difference between the presence of an infectious person and their contacts in the same place. However, in our case, the situation is unique because it included a small number of people who visited the venue for precisely scheduled activity (time and place were very well known). Additionally, activities were performed in specific circumstances (small room with poor ventilation in which players spend 45 min, intense physical activity, moist and warm atmosphere) at the very beginning of COVID-19 epidemic. These factors could explain why no similar event has been reported until now.

## Conclusion

Acquired data from our epidemiological investigation and scientific data that are available so far, raises possibility that transmission occurred indirectly through contaminated objects in the changing room or squash hall (doorknobs, clothes stands) or with virus aerosolisation in the squash hall.

Our findings advise caution in settings similar to observed. Acknowledging the possibility of presented transmission is especially important when implementing less strict preventive measures and reopening gyms or other indoor sport facilities, wellness centres and night clubs.

We emphasise the need for further research in modes of transmission in order to implement appropriate control measures to prevent nosocomial spread and superspreading events.

## Data availability statement

The data that support the findings of this study are available on request from the corresponding author (Z. Simonović). The data are not publicly available due to restriction regarding participants' privacy.
